# High efficiency CHO cell display-based antibody maturation

**DOI:** 10.1038/s41598-020-65044-7

**Published:** 2020-05-15

**Authors:** Ruiqi Luo, Yun Zhao, Yingjun Fan, Lili An, Tao Jiang, Shaohua Ma, Haiying Hang

**Affiliations:** 1 Key Laboratory for Protein and Peptide Pharmaceuticals, National Laboratory of Biomacromolecules, Institute of Biophysics, Chinese Academy of Sciences, Beijing, 100101 China; 2 University of Chinese Academy of Sciences, Beijing, 100039 China; 3 National Laboratory of Biomacromolecules, Institute of Biophysics, Chinese Academy of Sciences, Beijing, 100101 China; 4 Department of Thoracic Surgery, Peking University Third Hospital, Beijing, 100191 China

**Keywords:** Antibody generation, High-throughput screening

## Abstract

Previously, we developed a CHO cell display-based antibody maturation procedure in which an antibody (or other protein) gene of interest was induced to mutate by activation-induced cytidine deaminase (AID) and then form a library by simply proliferating the CHO cells in culture. In this study, we further improved the efficiency of this maturation system by reengineering AID, and optimizing the nucleic acid sequence of the target antibody gene and *AID* gene as well as the protocol for *AID* gene transfection. These changes have increased both the mutation rate and the number of mutation type of antibody genes by more than 10 fold, and greatly improved the maturation efficiency of antibody/other proteins.

## Introduction

Display technologies including phage, yeast, and bacteria displays have been used to mature antibodies for affinity and stability improvement. Each of these technologies has its advantages and disadvantages in library-constructing efficiency, ease of displaying diverse antibodies and speed to obtain desired clones^1–7^. In recent years, mammalian cell display has also been developed. Compared with the above described displays, mammalian cell display is advantageous in efficiently displaying diverse mutant clones, a high success rate of obtaining clones for subsequent mass production in CHO cells (a dominant way for therapeutic antibody/protein production) and posttranslational modifications. The last benefit is especially important for maturing extracellular domains of receptors (or ligands) against their corresponding ligands (or receptors). Many of these proteins are glycoproteins. However, transfection of plasmids into mammalian cells is not very efficient, and it is impossible to construct a sufficiently large library in this way. One way to circumvent the problem is to use viruses that carry antibody genes to efficiently infect mammalian cells^8^; another approach is to mutate antibody genes in cells by activation-induced cytidine deaminase (AID)^9,10^. However, in both ways mammalian cells often contain multiple antibody genes, making the identification of desired clones time-consuming. To overcome this difficulty, we transfected AID into mammalian cells that carry only a single antibody gene to mutate the antibody gene in cells during cell proliferation^11^, simply generating a mammalian cell carried antibody library by growing these cells in an incubator, a process much easier than the library construction in any other display technologies.

AID initiates somatic hypermutation (SHM) by converting deoxycytidines (dC) to deoxyuracils (dU) which then can induce other mutations, and plays a central role in introducing diversification of the antibody repertoire in B cells^12–14^. In this study, we intend to further improve the efficiency of AID-mediated CHO cell display by reengineering AID, and optimizing the nucleic acid sequence of the target antibody gene and the AID gene, as well as optimizing the protocol of AID gene transfection. Through these changes, we have enhanced both the mutation rate and the number of mutation type of antibody genes by more than 10 fold, and greatly increased the maturation efficiency of antibody/other proteins.

## Results

### Construction of a highly efficient AID for mutating a targeted gene

**Figure 1:**
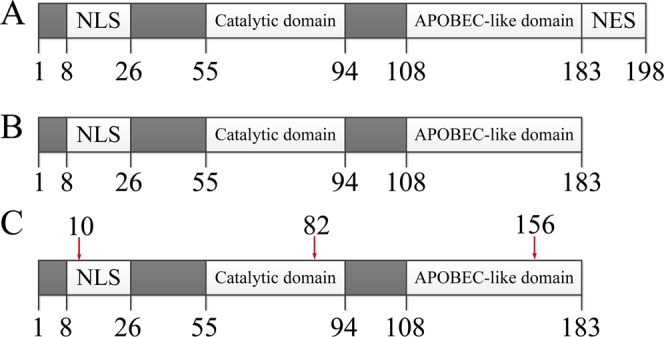
The changes made on mammalian AID for improving its activity. AID proteins include nuclear localization signal (NLS), nuclear export signal (NES), catalytic domain, and APOBEC protein-like domains. (**A**) The wild type AID structure (named AID); (**B**) The AID without NES (AID-del); (**C**) The AID-del containing K10E, T82I and E156G point mutations (AID-plus). The mouse and human AID’s (mAID and hAID) have the same basic structure. The numbers below the molecules are amino acid sequence numbers, and the numbers above the AID-plus molecule are the amino acid sequence numbers with point mutation.

In previous studies, many rounds (5 or more) of AID-induced mutation and flow cytometric sorting had to be carried out to achieve satisfactory affinity for the targeted antibodies^9–11^. We inferred that low AID enzyme activities were one of the reasons for the requirement of many rounds of mutation and sorting. It was reported that the removal of the nuclear output signal (NES) at the C-terminus of AID accumulated more of the AID in the nucleus, and increased the mutation rate on target genes^15^. In addition, AID mutants bearing the point mutations K10E, T82I and E156G have a higher catalytic activity^16^. We constructed a mouse AID enzyme mutant (mAID-plus) by removing NES and carrying the three point mutations (Fig. 1), and tested its mutation efficiency in CHO cells.

We used a *GFP* gene (GFP*) to compare the mutation efficiencies of mAID and mAID-plus. GFP* gene contains a stop codon in its coding region and is unable to be translated into fluorescent GFP, and only after reverse mutation takes place on the artificial stop codon, the GFP becomes fluorescent and the cells bearing it become fluorescent^17–19^.

**Figure 2:**
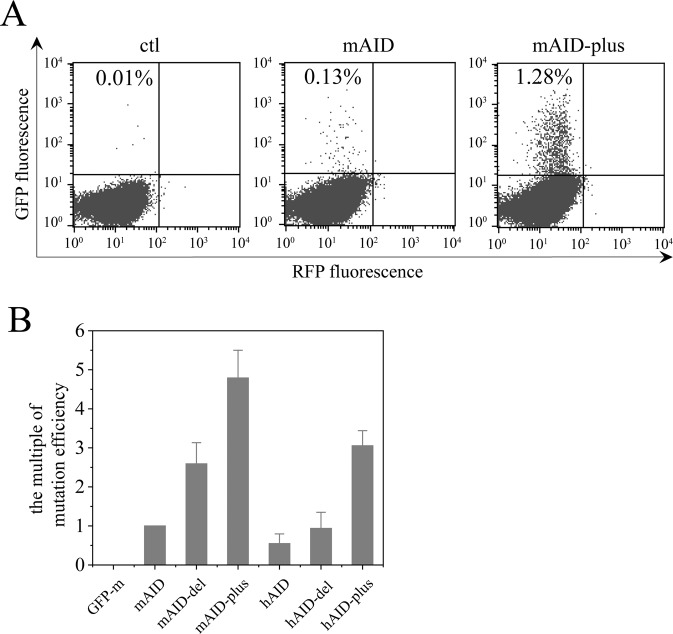
Comparison of the mutation efficiencies of different AID mutant molecules on a target GFP gene. (**A**) The GFP reporter gene bearing a stop codon was used to detect the mutation efficiency of mAID and mAID-plus. The ordinate indicates the fluorescent signal of GFP, the percentage of reverse mutants are shown in the figures. (**B**) The GFP reporter gene was used to detect mutation efficiency of different AID mutants. The mAID data was used as control, and the hAID, mAID-del, mAID-plus, hAID-del, and hAID-plus data were normalized to mAID to quantitatively compare the mutation efficiencies of the above AID’s.

In this study, *mAID* and *mAID-plus*, each cloned into the episomal expression plasmid pCEP4 were transfected separately into CHO cells with pcDNA3.1-GFP*. mAID-plus converted far more GFP* to *GFP* than mAID, indicating that mAID-plus has a much higher capacity to mutate GFP* (Fig. 2A). Further analyses showed that both the point mutations (K10E, T82I and E156G), and the deletion of mAID’s NES contributed to the improvement of mAID activity (Fig. 2B).

We also constructed hAID-del (human AID without NES) and hAID-plus (hAID-del with the point mutations K10E, T82Iand E156G) and tested their mutation efficiencies (Fig. 2B). Generally speaking, hAID and their mutants had lower activities than their mouse counterparts in CHO cells. The mutations K10E, T82I and E156G on hAID increased its activity, while in contrast to mAID, the NES deletion of hAID did not enhance its activity. These data suggest that mAID-plus has the highest mutating activity, and should be used for antibody affinity maturation in the following experiments.

### The contributions of the base optimization of target antibody gene and the engineered AID to mutation efficiency

**Figure 3:**
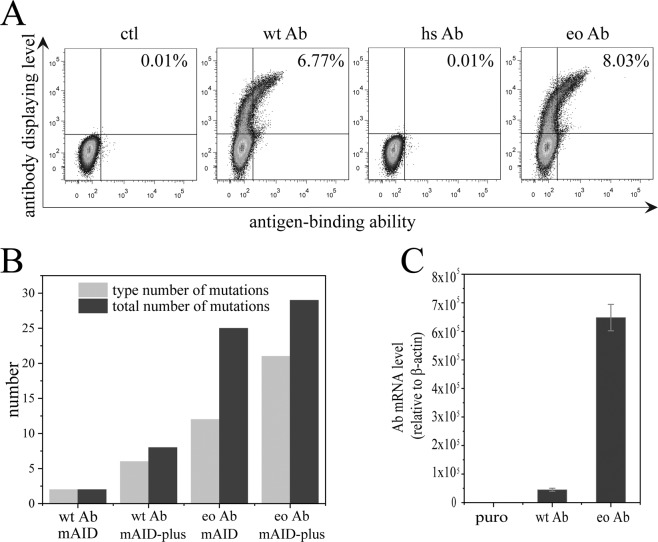
Comparison of the mutation efficiencies of different combinations of various AID’s and antibodies with different gene sequences. (**A**) Antibody display levels of cells transfected with 3 different TNFα antibody genes were detected 2 days after transfection. These 3 antibodies have the same amino acid sequence but different gene sequences; wtAb is the wild type antibody, hsAb is the antibody sequence-optimized for the highest content of AID mutation hot spots, and eoAb is the antibody sequence-optimized for the highest gene expression. Refer to Materials and Methods for the detailed description. (**B**) Affinity maturation evolution of TNFα antibodies with the same amino acid sequence but with different gene sequences using different AID’s. (**C**) The transcription level of wtAb and eoAb measured with RT-PCR.

In the previous section, the engineered AID (AID-plus) demonstrated a superior activity for converting a stop codon into an amino acid and forming a functional GFP gene. A previous study from Honjo’s research group found that the AID-induced mutation sites were predisposed to divide into “hot spots” and “cold spots” in B cells^17^. To convert an antibody gene sequence of interest into the one containing as many “hot spots” as possible without changing its amino acid residue sequence, we developed a computer algorithm and converted the variable regions of an anti-TNFα single chain antibody (scFv) (described in Materials and Methods) using this algorithm (S1). However, the converted antibody gene (hsAb) could hardly be displayed (Fig. 3A). Western blot analysis demonstrated that the hsAb did not express while the wtAb expressed normally in host cells (Fig. S1). We inferred that the converted nucleic acids impaired the gene’s transcription and/or translation. The lack of expression is not due to rare codons since we intentionally removed all rare codons in the sequences generated from the computer algorithm we developed. Therefore, we had the mutability optimized antibody gene processed using a computer program “OptimumGene”^20–22^ of the Genscript Biotech company to achieve a maximal expression of the gene (S1). Although this gene (eoAb) was highly expressed and displayed (S1 and Fig. 3A), we found that the computer program generated an identical antibody gene sequence whether it processed the original wild type gene or mutability optimized gene. That is, it erased all the base changes derived from our computer algorithm (S1), thus these two programs are incompatible.

We went ahead to check if the expression-optimization of the antibody gene could lead to a higher mutation efficiency. We paired mAID or mAID-plus with the wtAb or eoAb to investigate the contributions of the modifications of mAID and the sequence-optimization to inducing mutation on this scFv. A round of evolution is composed of (1) transfecting an mAID plasmid into CHO cells that displayed the antibody, (2) then proliferating the CHO cells in medium containing antibiotic to maintain the plasmid in cells, and (3) at last enriching the cells displaying the antibody at high levels as well as strongly binding to TNFα-GFP by flow cytometry sorting. After one round of evolution, the antibody genes isolated from the sorted CHO cells were cloned and sequenced. Fifty clones were sequenced for each sorted cell sample and at least 42 valid sequences were obtained from each of the four samples. The results showed that both the expression optimization and mAID modifications significantly increased mutation efficiency and the number of point mutation type (Table S1 and Fig. 3B). Notably, the types of point mutations from the four samples were hardly overlapping; among 36 different point mutations, only two point mutations (C525G, G726A) occurred in different samples. A single round of evolution had already enriched two point mutations from the two sequenced optimized samples (14 C525G mutations induced by mAID, 7 G371A mutations induced by mAID-plus, Table S1).

Obviously, the above expression-optimization mediated the enhancement in mutation rate and number of mutation type is not due to the introduction of new hot spots. The mutation efficiencies induced by AID was closely proportional to the transcription level of targeted genes^23^. We then checked if the expression-optimization program from Genscript Biotech increased antibody gene transcription. Indeed, the program increased transcription level by 14 fold as revealed by RT-PCR assay (Fig. 3C). This transcription enhancement contributed significantly more to the increase in both mutation rate and number of mutation type than that of mAID-plus. The ratios of mutation rate and number of mutation type of eoAb/mAID to wtAb/mAID are 12.5 and 6, respectively, while those of wtAb/mAID-plus to wtAb/mAID are only 4 and 3, respectively (calculated from Fig. 3B).

### Effect of the optimization of *AID* sequence for transcription on mutation efficiency of antibody gene

**Figure 4:**
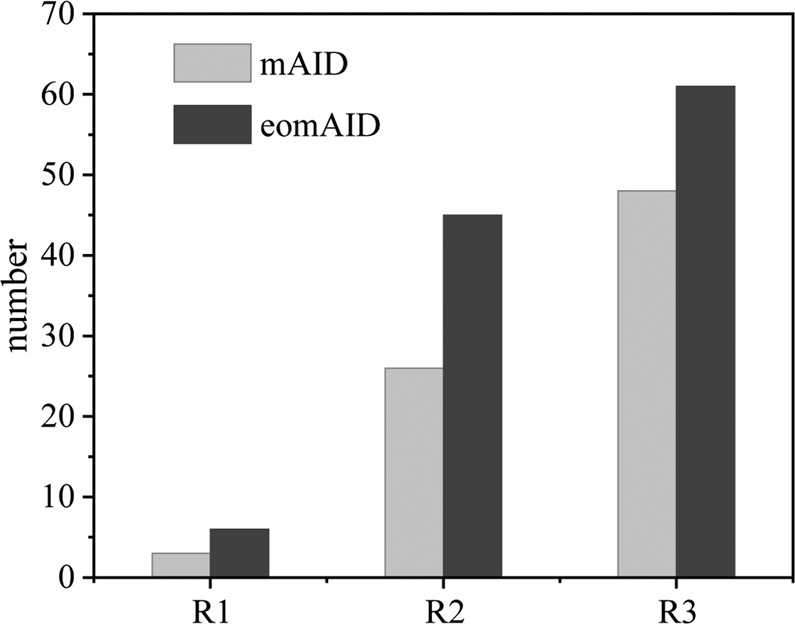
Comparison of mutation frequencies between mAID and optimized mAID (eomAID). Three rounds of the evolution were carried out. The total numbers of mutations were calculated for each round of evolution. R1, R2 and R3 stand for the results after Round 1, Round 2 and Round 3 evolution.

In the previous section, we showed that the expression-optimization of the scFv gene sequence for transcription enhanced its mutation rate. We were curious whether the expression-optimization of the AID gene sequence for transcription using the Genscript Biotech computer program would also enhance the mutation rate. We separately transfected with mAID and expression-optimized mAID (eomAID) into antibody-displayed cells and carried out 3 rounds of evolution. Fifty clones of each round of TNFα antibody gene were sequenced and point mutations identified (Table S2). In the cells derived from the first two rounds of evolution (R1, R2), the mutation frequency induced by the optimized mAID was about double that of the wild-type mAID; after the third round of evolution (R3), the mutation frequency induced by the optimized mAID was also significantly higher than that of the wild-type mAID (Fig. 4). The mutation frequency in the cells after the R2 round of evolution induced by the optimized mAID was nearly as high as that induced by the wild-type mAID after the R3 round of evolution.

### The effect of the transfection strategy of AID plasmids on the mutation efficiency of antibody gene

We demonstrated that the increased AID expression by AID expression-optimization enhanced the mutations of the targeted antibody gene in the above section. In the past, episomal vectors were used to express both antibody and AID genes for the reason that the episomal vector-bearing genes are not integrated into a chromosome and can then highly express the genes without the influence of a chromosomal location effect^9–11^. The high levels of AID and antibody gene transcription confer a high efficiency of antibody gene mutation^23^. Episomal vectors are thought to stay in the cytoplasm and are not integrated into chromosomes^24^. Thus, to conduct multiple rounds of antibody evolution, episomal vectors containing *AID* gene were transfected into mammalian cells displaying antibodies for each round of evolution followed by culturing the cells in medium containing antibiotic to keep the episomal vectors in cells^10,11^. To our surprise, however, the cells derived from 3 months’ culture without antibiotic after one round of evolution were still resistant to the antibiotic neomycin; the proliferation rate and morphology of these cells looked the same with or without neomycin in the medium. This indicates that the antibiotic-resistant gene *Neo* had already been integrated into chromosomes. Therefore, the newly transfected episomal vectors bearing *Neo* and *AID* genes after the first round of evolution cannot be kept in cells in medium containing neomycin, and the AID enzyme level should be lower than expected. In this case, the mutation rate of the antibody gene would be lower, resulting in inefficient antibody maturation.

**Figure 5:**
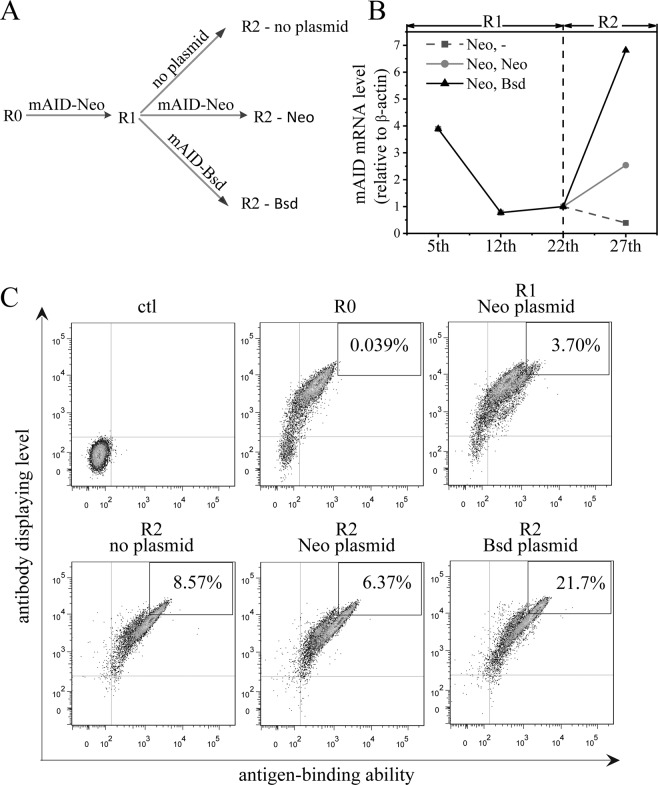
The effects of three different transfection strategies on AID expression and the accumulation of the cells displaying antibody mutants with improved antigen-binding abilities. (**A**) Procedure of the three transfection strategies. R0 is the CHO cell displaying wtAb, R1 and R2 stand for the cells derived from one and two rounds of maturation, respectively. No plasmid: no transfection; mAID-Neo: transfection with mAID-Neo plasmid; mAID-Bsd: transfection with mAID-Bsd plasmid. Refer to the text in Results and Materials and Methods for the details. (**B**) The AID mRNA levels in CHO cells using different transfection strategies. The first round of evolution was started with transfection with mAID-Neo plasmid. There are three different strategies for the second round of evolution: (1) no transfection, (2) transfection with mAID-Neo plasmid and (3) transfection with mAID-Bsd plasmid. The first (R1) and second round (R2) of evolution were started on day 0 and day 22, respectively. Cells were harvested on the 5th, 12th, 22nd and 27th day, respectively. The AID gene expression was detected by Real-time PCR. Three repetitive tests were carried out, and only one test result was presented here. (**C**) The percentages of cells with improved antigen-binding abilities, derived from three different transfection strategies. The cells included for the percentage calculation are only those with the highest display levels.

To avoid the undesired situation described above, we constructed a new episomal vector bearing the *AID* gene as well as the blasticidin-resistant gene (*Bsd*) (instead of *Neo*). We compared the AID mRNA levels among the 3 groups of cells: (A) the cells cultured 15 days without neomycin after one round of evolution (15 days after sorting); (B) the cells which were cultured 10 days without neomycin after sorting, then transfected with the episomal vector bearing the *AID* gene and *Neo*, and cultured 5 more days in the presence of neomycin; (C) the cells which were cultured 10 days without neomycin after sorting, then transfected with the episomal vector bearing the *AID* gene and *Bsd*, and cultured 5 more days in the presence of blasticidin (Fig. 5A). We measured the AID mRNA levels on the 5th, 12th (right before 1st sorting), 22nd (right before 2nd transfection; no 2nd transfection for the 1st group of cells) and 27th day (5 days after 2nd transfection) of the three groups of cells (Fig. 5B). In spite of the unstable experimental system, the data showed that transfection with the vector bearing *Bsd* yielded a much higher AID mRNA level than that without a 2nd transfection and that with a 2nd transfection with vector bearing *Neo*, and the AID mRNA level in the B group of cells was slightly higher than that of the A group. These results indicate that the repeated transfection with the vector containing the same antibiotic gene only confers an AID level slightly higher than that without the repeated transfection, and that the repeated transfection with a vector bearing a different antibiotic gene yields an AID level much higher than that of the repeated transfection with the vector containing the same antibiotic gene.

We wanted to know how the antibodies with the three transfection strategies evolved. We detected the display levels and antigen-binding abilities of the antibodies on the cells after two rounds of evolution as shown in Fig. 5C. The C group had more cells with the antigen-binding abilities above the wild type antibody than the A and B groups, and the cell numbers with the highest antigen-binding abilities of the groups A and B were about equal (Fig. 5C). Fifty clones of each group of TNFα antibody gene were sequenced and point mutations identified (Table S3). In the cells derived from the two rounds of evolution (R1, R2), the mutation frequency induced by group C was higher than that of the group A or B.

### Antibody affinity maturation

We demonstrated that the reengineered AID, the expression-optimized antibody gene, the expression-optimized AID gene, and an improved AID transfection strategy can enhance the mutations of the targeted antibody gene in the above section. In the past, to mature antibody affinity, the wild type AID was introduced once and the cells displaying antibody were put through multiple rounds of cell proliferation-flow sorting^9^, or the wild type AID was introduced into the cells displaying antibody once after each round of proliferation-flow sorting^11^. In this study, we compared these two maturation procedures with an evolution process in which AID-plus, expression-optimized antibody gene and the improved strategy were used. Specifically, the following three procedures were compared in their abilities to mature antibody affinity: (A) the cells displayed wtAb transfected with the episomal vector bearing the *AID* gene and *Neo* in the first round and enriched in the next two rounds; (B) the cells displayed wtAb transfescted with the episomal vector bearing the *AID* gene and *Neo* in the two rounds and enriched in the 3rd round; (C) the cells displayed eoAb transfescted with the episomal vector bearing the *AID-plus* gene and *Neo* in the first round, then transfected with the episomal vector bearing the *AID* gene and *Bsd* in the second round, and enriched in the 3rd round. Fifty clones of TNFα antibody gene derived from the cells after each round of evolution were sequenced and point mutations identified from the successfully sequenced genes (Table S4). We then choose six clones with the highest mutation frequency after the 3rd round of evolution (C344T, G371A, C720G, G721A, C74T + C720A, G81T + G721A, highlighted with colors in Table S4), and examined the affinities of these antibodies in the scFv form. All the mutant clones except G371A have higher affinities than the wild type. The G371A mutant antibody clone failed to express for unknown reason. The other five clones were analyzed by Octet biomolecular interaction technology for their dissociation coefficients (K_D_), confirming their improved affinities (Fig. S1, Table 1). We found that the two clones derived from the (C) procedure (C720G and C74T + C720A) had significantly higher affinities than those of the other three mutant clones derived from the (A) and (B) procedures.

**Table 1:** Affinities of different mutations.

Clone	Evolution group	Mutation	K_D_(M)	K_off_ (1/s)	K_on_ (1/MS)
WT	—	No	1.546E − 06	9.126E − 03	5.904E + 03
C344T	A or B	A115V	2.558E − 07	8.780E − 03	3.432E + 04
G721A	A or B	E241K	1.134E − 07	5.725E − 03	5.049E + 04
G81T + G721A	A or B	K27N + E241K	1.986E − 07	6.673E − 03	3.360E + 04
C720G	C	N240K	2.956E − 08	4.274E − 03	1.446E + 05
C74T + C720A	C	T25I + N240K	3.162E − 08	4.452E − 03	1.408E + 05

The values of K_on_ represented the association rates, the values of K_off_ represented the dissociation rates, and K_D_ = K_off_ /K_on_.

## Discussion

Compared to the widely used phage display and yeast display, CHO cell display possesses advantages in codon usage, posttranslational modification and ease in library construction^25^, but also has a disadvantage in the limited diversity of a target gene due to a low AID-induced mutation rate. In this study, we reengineered AID, optimized the antibody gene and AID sequences, and formatted a new AID transfection strategy. These changes led to enhancements of both the mutation rate and diversity by more than 10 fold (Fig. 3B), and to a highly efficient maturation procedure (Fig. 5C and Table 1).

AID activity is an important factor in inducing mutations in a gene of interest. We found that mouse AID has higher activity than human AID (Fig. 2B). We thought that this might be because mouse is closer to Chinese hamster than Human in systemic evolution. However, CHO AID has a lower activity than mouse AID (our unpublished data). Interestingly, the deletion of the nuclear export signal of human AID did not increase the gene mutation efficiency as demonstrated for mouse AID (Fig. 2B) although the point mutations of K10E, T82I and E156G in both mouse and human AIDs confer comparable activity enhancements; this phenomenon is beyond our understanding at present. Based on the two unique features of mouse AID, mAID-plus is the best choice for CHO cell display-based maturation. A recent study by Al-Qaisi *et al*.^26^ compared transient and stable transfection of wild type and its mutant (m7.3) AID’s, finding that the mutant induced significantly more mutations in the targeted genes, consistent with our results in this study in which the mAID-plus was actually an m7.3 with the deletion of the AID nuclear export signal (NES). This study presented data showing that the level of the mutations on the *RFP* gene reached a plateau 10 days after transient transfection of wild type or m7.3 AID into host cells. These data are also compatible with our results in which the AID mRNA level at the 5th day after *AID* transfection into host cells was much higher than that at 12th day (5 times higher, Fig. 5B).

The transcription level of a targeted gene is positively proportional to the rate of AID-induced mutation on this gene^23^. This was confirmed by optimizing the TNFα antibody gene sequence for the best expression using the computer program of the Genscript Biotech Company. The increase in expression is partially due to an improvement in transcription or translation (Fig. 3C).

An episomal vector was thought not to be integrated into cellular chromosomes and used to highly express the gene on the vector due to the lack of location-effects on the gene expression^9,25^. However, our tests showed that the gene on the vector including the antibiotic-resistant gene was integrated in chromosomes after just one round of evolution. The procedures in which an episomal vector bearing an identical antibiotic-resistant gene is repeatedly transfected into the same cells for antibody maturation cannot be kept in the cells by the corresponding antibiotic. Our data demonstrated that transfecting the cells with the vectors containing different antibiotic resistant genes besides AID and culturing cells with their corresponding antibiotics in medium for different rounds of evolution maintained high levels of AID enzyme in cells, and increased the mutation efficiency of the gene of interest. A recent study by Liu LD *et al*.^27^ screened engineered deaminases and CRISPR-deaminase coupling approaches and built diversifying base editors to generate SHM. At the same time, Devilder MC *et al*.^28^ use the CRISPR-Cas 9 to target AID to antibody genes. However, these systems rely on the mammalian cells endogenous DNA repair system, which potentially limits its efficiency or usage.

The types of point mutations from the four samples were barely overlapping; among 36 different point mutations, only two point mutations occurred in different samples (Table S1). This rarity in overlapping point mutations is not only due to the difference in target gene sequences (original versus optimized), but also due to different AID enzymes (mAID versus mAID-plus); only one mutation took place in both samples among 29 different point mutations in the cells that possessed the optimized antibody gene sequence and were mutated respectively by mAID and mAID-plus. These results indicate that the preferred target base sequences of mAID-plus is very different from those of mAID. Notably, the combination of mAID and the optimized antibody sequence yielded a significantly higher mutation rate and diversity than those derived from the combinations of the wild type sequence and mAID or mAID-plus (Table S1), although its levels of mutation rate and diversity were not as high as those resulting from the combination of the optimized sequence and mAID-plus (Fig. 3B). Based on this analysis, it is preferable to use mAID and mAID-plus in different rounds of evolution to increase the opportunity to enrich the desired combinations of point mutations.

The aforementioned changes have made the CHO cell-based evolution procedure highly efficient. In the past, Bowers *et al*. used a human cell-display based evolution procedure to mature antibody affinity in which the wild type AID was introduced once and the cells displaying antibody were put through multiple rounds of cell proliferation-flow sorting^9^. Our laboratory used a similar procedure, but in which the wild type AID was introduced into the CHO cells displaying antibody once after each round of proliferation-flow sorting^11^. To show the values in antibody affinity maturation of the elements examined in this study (modified AID, expression-optimized AID as well as antibody gene sequences, and transfection strategy), we formatted an affinity maturation procedure in which an example set of changes on these elements were made, and compared the efficiency of this procedure with those of the two above-mentioned previous maturation procedures. Three rounds of evolution using the newly formatted procedure generated two affinity-improved antibodies (N240K and T25I + N240K), and 3 rounds of evolution using the two old procedures produced three affinity-improved antibodies (A115V, E241K and K27N + E241K). The affinities of the two antibodies derived from the new procedure are 3.5 to 8.6 higher than those of the three antibody clones generated from the two old methods (calculated from the data in Table 1), suggesting the superiority of the new procedure to the old ones.

Based on the results in this study, the ideal procedure should be: (1) transfection with a plasmid containing Neo and mAID-plus before the first flow sorting, and (2) transfection with another plasmid bearing Bsd and mAID before the second flow sorting. Of course, all the 4 genes can be paired differently such as Neo and mAID as well as Bsd and mAID-plus, and transfection sequences can be rearranged. This procedure is most likely to produce the highest mutation rate and the highest mutation diversity, and acquire the best mutation combinations conferring the most desired antibody features. We expect that this procedure will be more frequently used in maturing antibodies and other proteins in the future.

## Materials and Methods

### Plasmid construction and expression

TNFα-GFP is His-tagged on its N-terminus and was prepared by following the procedure by Chen *et al*.^19^. Briefly, His-TNFα-GFP was synthesized in *E. coli* containing the PET28a (+)-*TNFα-GFP* plasmid, was purified with a Ni column. The concentration of the prepared TNFα-GFP was 2 mg/mL.

Anti-human TNFα wild type scFv (αTNFα) is from Jie Tang (Institute of Biophysics, Chinese Academy of Sciences, Beijing, China).The plasmids pF2AC for co-expression of Flpo and iCre recombinases, pFAbL for the replacement of the puro with the antibody (αTNFα antibody) gene in a predetermined genomic locus of CHO-puro cells were described in detail previously^11^.

The exchange vector pFRT-αTNFα-loxP was generated previously in our laboratory^11^, and for the current study pFRT-eo-αTNFα-loxP was created by replacing SP-HA-anti-TNFα single chain antibody gene-TM (SP-HA: signal peptide-HA tag) with SP-HA-eo-anti-TNFα single chain antibody gene-TM (the gene sequence was optimized for the best expression by the Genscript Biotech Company (Nanjing, China)) between *Bam*HI and *Bgl*II in pFRT-αTNFα-loxP.

The pCDNA3.1(+)-GFP* plasmids were constructed by Chen *et al*.^19^.

Eight pCEP4-Ig-Ek plasmids expressing various AIDs were used in this study. The AIDs include mouse AID (mAID), mAID with its C-terminal nucleic export signal (NES) deleted (mAID-del), mAID-del bearing K10E, T82I and E156G (mAID-plus), and mAID as well as mAID-plus with their sequences optimized for the best expression (eomAID as well as eomAID-plus). The human AIDs are hAID, hAID-del and hAID-plus. The plasmid expressing mAID was described previously^11^. The DNA sequences of mAID-plus, eomAID, eomAID-plus, hAID and hAID-plus were synthesized by the Genscript Biotech Company. The DNA sequences of mAID-del and hAID-del were synthesized by performing PCRs using mAID and hAID as templates. The PCR primer pairs for the 7 AIDs contained *Hin*dIII and *Xho*I restriction sites, and the PCR products were cut with synthesized by *Hin*dIII and *Xho*I. The purified fragments were inserted between the *Hin*dIII and *Xho*I restriction sites in pCEP4-Ig-Ek plasmid.

Two pCEP4-Ig-Ek-mAID plasmids expressing *neomycin* and *blasticidin* resistant gene (*Bsd* and *Neo*) respectively were used in this study. The plasmid expressing *Neo* (pCEP4-Ig-Ek-Neo-mAID) was described previously^11^. pCEP4-Ig-Ek-Bsd-mAID was created by replacing *Neo* with *Bsd* in pCEP4-Ig-Ek-Neo-mAID between *Age*I and *Not*I. *Bsd* was synthesized by the Genscript Biotech Company.

### Cell culture

CHO/dhFr cells (12200036) were purchased from The Cell Bank of the Chinese Academy of Sciences, Shanghai, China, and cell lines derived from CHO/dhFr^−^ were propagated in IMDM medium (HyClone, USA) containing 10% fetal bovine serum (HyClone, USA), 0.1 mM hypoxanthin, and 0.016 mM thymidine (Gibco, USA), at 37 °C in a 5% CO_2_ incubator. Subcultures were carried out every 2–3 days. CHO-puro cells (The CHO/dhFr^−^ cells with a single copy of retargetable high-level expression cassette) were established previously and described in detail^11^.

### Transfection and antibody affinity maturation

To test the mutant ability of the eight pCEP4-Ig-Ek plasmids expressing mAID, mAID-del, mAID-plus, hAID, hAID-del and hAID-plus, CHO-puro cells were seeded into each well of a six-well plate. Cells were transfected with a mixture of 1 μg pCDNA3.1(+)-GFP*, 1 μg pCEP4-Ig-Ek plasmids expressing various AIDs, and 5 μL Lipofectamine^TM^ 2000 (Invitrogen, USA) for 6 h, separately. After 48 h, cells were analyzed using a FACSCalibur (BD) flow cytometer.

Generally, to replace the antibody gene integrated in chromosome in CHO cells, CHO-puro cells were seeded into each well of a six-well plate. Cells were transfected with a mixture of 0.5 μg exchange vector, 2 μg pF2AC, and 5μL Lipofectamine^TM^ 2000 (Invitrogen, USA) for 6 h. After transfection, the cells were transferred into a 10 cm dish containing IMDM medium with 10% FCS. Afterwards, the cells were collected and incubated with PE-conjugated anti-HA antibody (Abcam,1:250 in cold opti-MEM medium [Invitrogen, USA], to detect antibody display levels.) for 30 min at 4 °C, washed with cold opti-MEM once, resuspended in cold opti-MEM, and sorted for cells that expressed high displaying antibodies using a FACSAriaIII (BD) flow cytometer.

To mature antibody affinity, CHO-puro cells which displayed anti-TNFα antibodies were seeded into a six-well plate. The cells were transfected with 2 μg of pCEP4-Neo-AID and 5 μL of Lipofectamine^TM^ 2000 for 6 h, washed and maintained in IMDM containing 10% FCS and HT for one day, then the cells were expanded in IMDM with 10% FCS, HT, 1 mg/mL neomycin for 11 days and incubated with PE-conjugated anti-HA antibody and TNFα-GFP (1:10000 in cold opti-MEM medium [Invitrogen, USA], to detect antigen binding ability.) for 30 min at 4 °C, and sorted for cells that expressed high affinity antibodies using a FACSAriaIII (BD) flow cytometer.

### Detection of antibody mutations

The genomic DNA of sorted RMCE-Ab cells was purified with a genomic DNA purification system (Promega). The antibody gene fragments were amplified from the genomic DNA and cloned into pMD-19 T-vector. Primers used for antibody gene PCR were as follows:

CMV-F: CGCAAATGGGCGGTAGGCGTG;

TM-R: CTGCGTGTCCTGGCCCACAGC.

### Quantitative real-time PCR analysis

Total RNA was isolated with RNeasyMini Kit (Qiagen) following the manufacturer’s protocol. For reverse transcription-PCR (RT-PCR), 2 μg of total RNA was reverse transcribed in a reaction volume of 20 μl to form cDNA using the SuperScript First-Strand Synthesis System (Invitrogen). Real-time PCR was performed using the StepOnePlus system (ABI) with SYBR Green I (Takara) to label amplified DNA. A standard curve method of quantification was used to calculate the expression of target genes relative to the housekeeping gene β-actin. Experiments were performed three times. The following primer pairs were used for the PCR reactions:

mAID- F1:GGCCACCTTCGCAACAAG;

mAID- R1:AGTCTCCGCAGCCCCTCA;

β-actin- Chinese hamster -F2:GGCCAACCGTGAAAAGATGA;

β-actin- Chinese hamster -R2:CGACCAGAGGCATACAGGGAC.

PCR procedures for these genes were template denaturation at 95 °C for 10 min, then 40 cycles of 95 °C for 10 sec, 60 °C for 30 sec, and a final extension at 72 °C for 3 min.

### Antibody affinity measurement

The affinities of various antibodies were measured by the Octet biomolecular interaction technology (ForteBio Octet, Menlo Park, CA, USA). TNFα-GFP protein were conjugated with biotin according to the manufacturer (ForteBio Octet), and concentrated to 50 μg/mL. The concentrated antigen was added and fixed to Streptavidin (SA) biosensors. The preparation of the antibody proteins were described above. The detection conditions used were (1) baseline 240 s; (2) loading 600 s; (3) baseline 180 s; (4) association 120 s with a series of concentrations (8000 nM, 4000 nM, 2000 nM, 1000 nM, 500 nM, 250 nM for wild type antibody; 2000 nM, 1000 nM, 500 nM, 250 nM, 125 nM, 62.5 nM for C344T, G721A and G81T + G721A antibody; 250 nM, 125 nM, 62.5 nM, 31.25 nM, 15.62 nM, 7.81 nM for C720G and C74T + C720A antibody.) of αTNF-α scFv; (5) dissociation 180 s. The K_on_ and K_off_ rates were measured by Octet software and K_D_ was calculated for each antibody mutation by the K_off_/K_on_ ratio.

## Supplementary information

Supplementary information

## Data Availability

The data sets generated during and/or analyzed during the current study are available from the corresponding author upon reasonable request.
