# Supplementary material for: High efficiency CHO cell display-based antibody maturation
Source: Sci Rep. 2020 May 15;10:8102. doi: 10.1038/s41598-020-65044-7 (PMC7229201; doi:10.1038/s41598-020-65044-7)
Supplement: Supplementary file 1 — Supplementary information [file 41598_2020_65044_MOESM1_ESM.docx]

**Supplementary Information**

**High efficiency CHO cell display-based antibody maturation**

Ruiqi Luo^1,2^, Yun Zhao^1^, Yingjun Fan^1,2^, Lili An^1^, Tao Jiang^2,3^, Shaohua Ma^4*^, Haiying Hang^1*^

1. Key Laboratory for Protein and Peptide Pharmaceuticals, National Laboratory of Biomacromolecules, Institute of Biophysics, Chinese Academy of Sciences, Beijing 100101, China

2. University of Chinese Academy of Sciences, Beijing 100039, China

3. National Laboratory of Biomacromolecules, Institute of Biophysics, Chinese Academy of Sciences, Beijing 100101, China

4. Department of Thoracic Surgery, Peking University Third Hospital, Beijing, 100191, China

* Corresponding author.

Haiying Hang. Tel/Fax: +86-10-64888473, E-mail: hh91@ibp.ac.cn. Correspondence may also be addressed to Shaohua Ma. Tel/Fax: +86-10-82267301, Email: [doctormsh@163.com](mailto:doctormsh@163.com).

S1. Nucleic acid sequence of the three different anti-TNFα antibodies.

wtAb GGAATTAATTCGGATCCCAATTCGCAGATCCAGCTGGTGCAGTCTGGACC

hsAb GGAATTAACTCGGATCCCAACAGCCAGATCCAGCTGGTTCAGTCTGGACC

eoAb GGAATCAATAGCGACCCAAACTCACAGATCCAGCTGGTGCAGAGCGGACC

***** ** : ** **.** : ************** ***: *****

wtAb TGAGCTGAAGAAGCCTGGAGAGACAGTCAAGATCTCCTGCAAGGCTTCTG

hsAb TGAGCTGAAGAAGCCTGGAGAGACAGTTAAGATCAGTTGCAAGGCTTCTG

eoAb TGAACTGAAAAAACCCGGAGAAACCGTCAAAATTAGTTGCAAGGCATCCG

***.*****.**.** *****.**.** **.** : ********:** *

wtAb GGTATACCTTCACAAACTATGGAATGAACTGGGTGAAGCAGGCTCCAGGA

hsAb GGTATACCTTCACAAACTATGGTATGAACTGGGTTAAGCAGGCTCCAGGT

eoAb GGTACACCTTCACAAACTATGGAATGAATTGGGTGAAACAGGCTCCAGGC

**** *****************:***** ***** **.***********

wtAb AAGGGTTTAAAGTGGATGGGCTGGATAAACACCTACACTGGAGAGCCAAC

hsAb AAGGGTTTAAAGTGGATGGGCTGGATAAATACCTATACTGGAGAGCCAAC

eoAb AAGGGGCTGAAATGGATGGGCTGGATTAATACTTACACCGGGGAGCCCAC

***** *.**.**************:** ** ** ** **.*****.**

wtAb ATATGCTGATGACTTCAAGGGACGGTTTGCCTTCTCTTTGGAAACCTCTG

hsAb TTATGCTGATGACTTCAAGGGACGGTTTGCCTTCAGTTTGGAAACCTCTG

eoAb TTATGCTGACGATTTCAAGGGGCGGTTCGCATTTTCTCTGGAAACTAGTG

:******** ** ********.***** **.** : * ******* : **

wtAb CCAGCACTGCCTATTTGCAGATCAACAACCTCAAAAATGAGGACTCGGCT

hsAb CCAGTACTGCCTACTTGCAGATCAACAACCTCAAAAATGAGGACTCGGCT

eoAb CCTCAACCGCTTACCTGCAGATCAACAATCTGAAAAACGAGGACAGCGCC

**: ** ** ** ************* ** ***** ******: **

wtAb ACATATTTCTGTGCAGGAAGAAGAAGCTATGATTACGACGTGGCTATGGA

hsAb ACTTACTTCTGTGCTGGTAGAAGAAGCTATGATTACGACGTGGCTATGGA

eoAb ACATATTTTTGCGCTGGCCGGAGATCCTACGACTATGATGTGGCCATGGA

**:** ** ** **:** .*.***: *** ** ** ** ***** *****

wtAb CTACTGGGGTCAAGGAACCTCAGTCACCATCTCCTCAGGATCTGGAAGTT

hsAb CTACTGGGGGCAAGGTACCTCAGTTACCATCAGTTCAGGTTCTGGTAGTT

eoAb TTACTGGGGACAGGGCACCAGCGTCACAATCAGCTCCGGGTCAGGATCTA

******** **.** ***: .** **.***: **.** **:**:: *:

wtAb CAGGAAGCGGCTCAAGTGGGTCTGGAAGCTCAGACATTGTGCTCACCCAG

hsAb CAGGTAGCGGCAGTAGTGGTTCTGGTAGCTCAGACATTGTGCTTACCCAG

eoAb GTGGCAGCGGGTCAAGCGGATCCGGCTCCTCTGACATTGTGCTGACACAG

:** ***** : :** ** ** ** : ***:*********** **.***

wtAb TCTCCAGCTTCTTTGGCTGTGTCTCTAGGGCAGAGGGCCACCATATCCTG

hsAb AGCCCAGCTAGTTTGGCTGTGAGCCTAGGGCAGAGAGCTACCATAAGTTG

eoAb AGCCCAGCATCCCTGGCCGTCTCTCTGGGACAGAGGGCAACTATCAGTTG

: *****:: **** ** : **.**.*****.** ** **.: **

wtAb CAGAGCCAGTGAAAGTGTTGATAGTTATGGCAATTATTTTATGCACTGGT

hsAb CAGAGCCAGTGAAAGTGTTGATAGTTATGGCAACTACTTTATGCACTGGT

eoAb TCGCGCCAGCGAATCCGTGGATAGCTACGGAAACTACTTCATGCACTGGT

.*.***** ***: ** ***** ** **.** ** ** **********

wtAb ATCAGCAGAAACCAGGACAGCCACCCAAACTCCTCATCTATCGTGCATCC

hsAb ACCAGCAGAAACCAGGGCAGCCACCCAAACTACTCATCTACAGAGCTAGT

eoAb ACCAGCAGAAGCCTGGCCAGCCCCCTAAACTGCTGATCTATCGAGCCTCT

* ********.**:** *****.** ***** ** ***** .*:** :

wtAb AACCTAGAATCTGGGATCCCTGCCAGGTTCAGTGGCAGTGGGTCTGGGAC

hsAb AACCTAGAATCTGGTATACCTGCCAGGTTCAGTGGCAGTGGTTCTGGTAC

eoAb AATCTGGAAAGTGGCATTCCTGCTAGATTCTCTGGGAGTGGATCAGGCAC

** **.***: *** ** ***** **.***: *** ***** **:** **

wtAb AGACTTCACCCTCACCATTAATCCTGTGGAGGCTGATGATGTTGCAACCT

hsAb AGACTTTACCCTTACCATTAACCCTGTGGAGGCTGATGATGTAGCTACCT

eoAb CGACTTTACACTGACTATTAACCCAGTGGAGGCTGACGATGTCGCAACAT

.***** **.** ** ***** **:*********** ***** **:**.*

wtAb ATTACTGTCAACAAAGTAATGAGGAGCCTCTCACGTTCGGCTCGGGGACA

hsAb ACTACTGCCAGCAAAGTAATGAGGAGCCTCTCACGTTCGGCTCGGGTACA

eoAb ACTATTGTCAGCAGAGCAACGAGGAGCCCCTGACATTCGGCAGCGGGACC

* ** ** **.**.** ** ******** ** **.******: ** **.

wtAb AAGTTGGAAATAAAACGG

hsAb AAGTTGGAAATAAAACGG

eoAb AAACTGGAAATCAAGCGG

**. *******.**.***

Figure S1


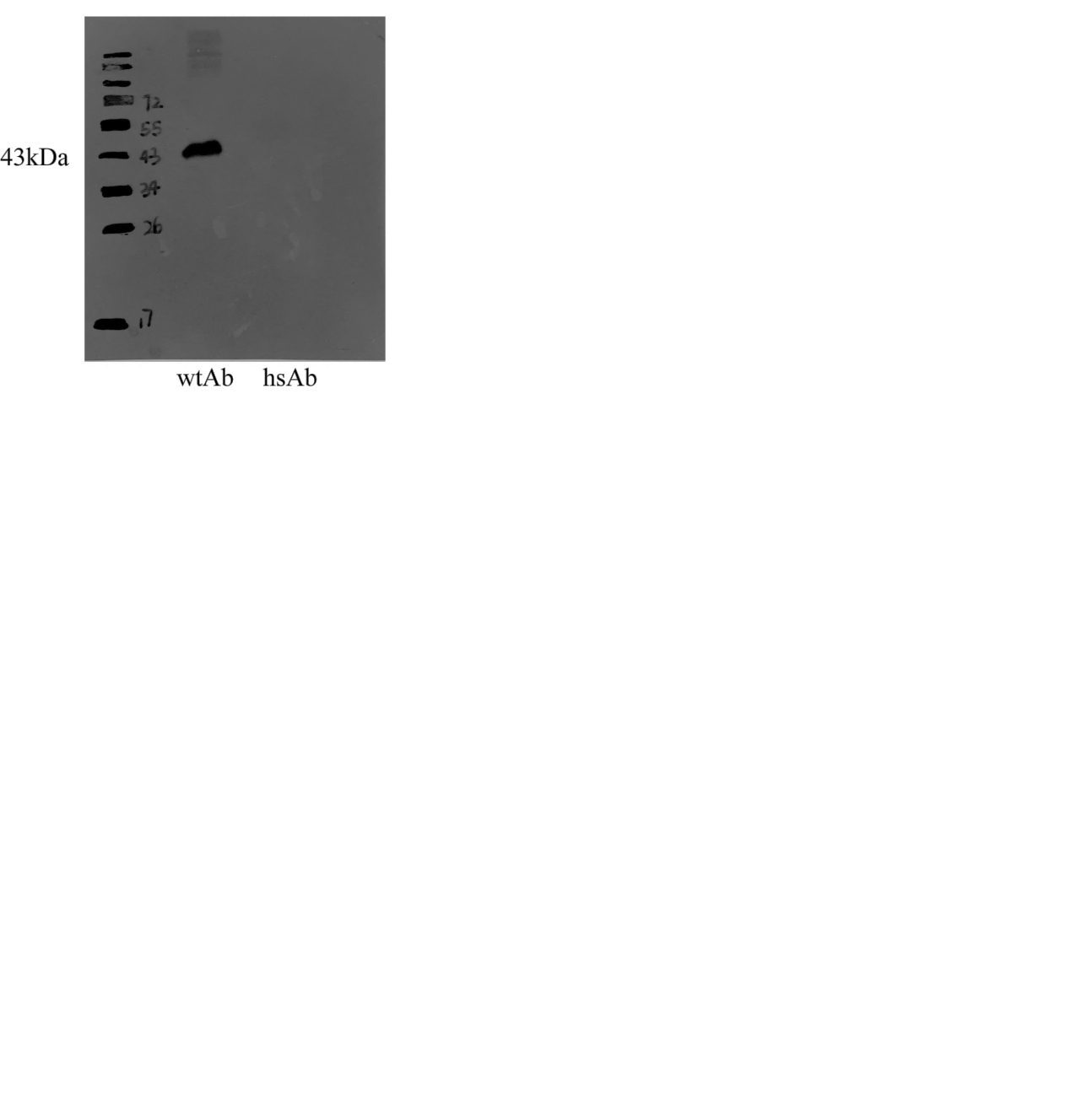


Figure S1 Western blot analysis of the antibody.

Table S1 Mutations observed during the affinity maturation procedure

| mutation | wtAb | | eoAb | |
| --- | --- | --- | --- | --- |
|  | mAID | mAID-plus | mAID | mAID-plus |
| C30T |  |  |  | 1/47 |
| A194G |  |  |  | 1/47 |
| C196T |  | 2/42 |  |  |
| C251T |  |  |  | 1/47 |
| C318T |  |  |  | 1/47 |
| C344T | 1/48 |  |  |  |
| G371A |  |  |  | 3/47 |
| T399C |  |  |  | 1/47 |
| A480G |  |  | 1/45 |  |
| T494C |  |  |  | 1/47 |
| G511A |  |  | 2/45 |  |
| C525G |  |  | 10/45 | 1/47 |
| G543T |  |  | 1/45 |  |
| C546G |  | 1/42 |  |  |
| C556G |  |  |  | 1/47 |
| C562T |  |  |  | 2/47 |
| T612C |  |  | 1/45 |  |
| T708G | 1/48 |  |  |  |
| G724A |  | 2/42 |  |  |
| G726A |  | 1/42 |  | 1/47 |
| A75G  G595A |  | 1/42 |  |  |
| C95T  G298A |  |  |  | 1/47 |
| G149A  T207C |  |  |  | 1/47 |
| G149A  G371A |  |  |  | 1/47 |
| C157T  G371A |  |  |  | 1/47 |
| G371A  A594G |  |  |  | 1/47 |
| G456A  C525G |  |  | 1/45 |  |
| C525G  C556T |  |  | 1/45 |  |
| A675G  A701G |  |  |  | 1/47 |
| G363T  G371A  C405T |  |  |  | 1/47 |
| C525G  T708G  G726A |  |  | 1/45 |  |
| G638A  G681A  G694C |  |  | 1/45 |  |
| the type of mutations | 2/48 | 6/42 | 12/45 | 21/47 |
| the total number of mutations | 2 | 8 | 25 | 29 |
| the total number of bases (bp) | 36864 | 32256 | 34560 | 36096 |
| Frequency per 10^4^ | 0.54 | 2.48 | 7.23 | 8.03 |

the type of mutations: 2/48 represents 2 mutant cells in 48 successfully sequenced clones;

the total number of bases: represents the total number of bases corresponding to the target gene in the successfully sequenced clones, eg: 48×768bp=36864bp;

Frequency per 10^4^: the total number of mutations/ the total number of bases

CDRs are highlighted in colors (HC-CDR1, HC-CDR2, HC-CDR3, LC-CDR1, LC-CDR2, LC-CDR3).

Table S2 Mutations observed during the affinity maturation procedure

| mutation | mAID | | | eomAID | | |
| --- | --- | --- | --- | --- | --- | --- |
|  | S1 | S2 | S3 | S1 | S2 | S3 |
| C18T | 1/44 |  |  |  |  |  |
| T45G |  |  |  | 1/50 |  |  |
| T109A |  |  |  |  |  | 1/38 |
| A123C |  | 1/44 |  | 1/50 |  |  |
| C230T |  |  |  | 1/50 |  |  |
| C251T |  |  |  | 1/50 |  |  |
| G316A |  |  |  |  | 22/43 | 3/38 |
| C344T |  | 16/44 | 33/41 |  | 7/43 | 15/38 |
| G407A |  |  |  | 1/50 |  |  |
| G441A |  | 1/44 |  |  |  |  |
| G480A |  |  |  |  | 1/43 |  |
| G500A |  |  |  |  |  | 1/38 |
| G511A |  |  |  |  |  | 1/38 |
| C727T |  |  |  |  | 7/43 |  |
| C728T |  |  |  |  | 1/43 |  |
| A733C |  |  |  | 1/50 |  |  |
| G27A  G316A |  |  |  |  | 1/43 |  |
| A32G  C461G |  | 1/44 |  |  |  |  |
| C39T  C344T |  | 1/44 |  |  |  |  |
| G195C  G316C |  |  |  |  |  | 1/38 |
| G195C  C344T |  |  |  |  |  | 1/38 |
| A212G  G511A |  |  |  |  |  | 1/38 |
| G219T  C344T |  |  | 1/41 |  |  |  |
| G316A  G511A |  |  |  |  |  | 1/38 |
| G316A  G724A |  | 1/44 |  |  |  |  |
| C344T  G348T |  |  | 1/41 |  |  |  |
| C344T  C442T |  |  | 1/41 |  |  |  |
| C344T  G500A |  | 1/44 |  |  |  |  |
| G441A  G466A |  |  | 1/41 |  |  |  |
| C506T  C544T | 1/44 |  |  |  |  |  |
| C727T  C728T |  |  |  |  | 1/43 |  |
| G138A  G195C  G316C |  |  |  |  |  | 6/38 |
| G138A  G195C  C344T |  |  |  |  |  | 1/38 |
| G195C  T281C  G316A |  |  |  |  |  | 1/38 |
| A269G  C344T  C596G |  |  | 1/41 |  |  |  |
| G364A  C727T  C728T |  |  |  |  | 1/43 |  |
| C34T  G138A  G195C  G316C |  |  |  |  |  | 1/38 |
| T43C  G138A  G195C  C344T |  |  |  |  |  | 1/38 |
| T385C  G441A  G466A  G480A |  |  | 1/41 |  |  |  |
| the type of mutations | 3/44 | 8/44 | 10/41 | 6/50 | 7/43 | 11/38 |
| the total number of mutations | 3 | 26 | 48 | 6 | 45 | 61 |
| the total number of bases (bp) | 33792 | 33792 | 31488 | 38400 | 33024 | 29184 |
| Frequency per 10^4^ | 0.89 | 7.69 | 15.24 | 1.56 | 13.63 | 20.90 |

the type of mutations: 2/44 represents 2 mutant cells in 44 successfully sequenced clones;

the total number of bases: represents the total number of bases corresponding to the target gene in the successfully sequenced clones, eg: 44×768bp=33792bp;

Frequency per 10^4^: the total number of mutations/ the total number of bases

S1、S2、S3 respectively represent 3 rounds of evolution.

CDRs are highlighted in colors (HC-CDR1, HC-CDR2, HC-CDR3, LC-CDR1, LC-CDR2, LC-CDR3).

Table S3 Mutations observed during the affinity maturation procedure

| mutation | Rounds of evolution | | | |
| --- | --- | --- | --- | --- |
|  | R1 | R2 | | |
|  |  | Group A | Group B | Group C |
| C55T |  |  | 1/47 |  |
| C255T |  | 1/41 |  |  |
| C344T | 5/41 | 1/41 | 2/47 | 3/39 |
| G500A |  |  | 1/47 |  |
| G646A | 2/41 |  |  |  |
| G721A |  | 33/41 | 35/47 | 18/39 |
| T9A  G721A |  |  |  | 1/39 |
| G37A  G721A |  |  | 1/47 |  |
| G54A  C344T |  |  |  | 1/39 |
| G54A  G721A |  |  |  | 2/39 |
| C55T  G721A |  |  | 1/47 | 2/39 |
| C64T  G721A |  | 1/41 |  |  |
| C117G  G721A |  |  |  | 1/39 |
| T172G  G721A |  | 1/41 |  |  |
| C230G  G721A |  | 1/41 |  |  |
| C296T  G721A |  |  |  | 1/39 |
| C344T  G721A |  | 1/41 |  |  |
| T393C  G721A |  |  | 1/47 |  |
| G593A  G721A |  |  |  | 2/39 |
| C623T  G721A |  |  |  | 1/39 |
| G721A  A758G |  |  | 1/47 |  |
| G33T  G36A  G721A |  |  |  | 1/39 |
| G33T  C55T  G721A |  | 1/41 |  |  |
| C55T  G193A  G195A  G721A |  |  |  | 1/39 |
| the type of mutations | 2/41 | 8/41 | 7/47 | 13/39 |
| the total number of mutations | 7 | 46 | 47 | 50 |
| the total number of bases (bp) | 31488 | 31488 | 36096 | 29952 |
| Frequency per 10^4^ | 2.22 | 14.61 | 13.02 | 16.69 |

the type of mutations: 7/41 represents 7 mutant cells in 41 successfully sequenced clones;

the total number of bases: represents the total number of bases corresponding to the target gene in the successfully sequenced clones, eg: 41×768bp=31488bp;

Frequency per 10^4^: the total number of mutations/ the total number of bases

R1、R2 respectively represent 2 rounds of evolution.

CDRs are highlighted in colors (HC-CDR1, HC-CDR2, HC-CDR3, LC-CDR1, LC-CDR2, LC-CDR3).

Table S4 Mutations observed during the affinity maturation procedure

|  | wt Ab | | | | | | eo Ab | | |
| --- | --- | --- | --- | --- | --- | --- | --- | --- | --- |
|  | Group A | | | Group B | | | Group C | | |
|  | R1(Neo-mAID) | R2(-) | R3(-) | R1(Neo-mAID) | R2(Neo-mAID) | R3(-) | R1(Neo-mAID-plus) | R2(Bsd-mAID) | R3(-) |
| C30T |  |  |  |  |  |  | 1/47 |  |  |
| C55T |  |  |  |  | 1/47 |  |  |  |  |
| C74T |  |  |  |  |  |  |  |  | 2/39 |
| A194G |  |  |  |  |  |  | 1/47 |  |  |
| C251T |  |  |  |  |  |  | 1/47 |  |  |
| C255T |  | 1/41 |  |  |  |  |  |  |  |
| C318T |  |  |  |  |  |  | 1/47 |  |  |
| C344T | 5/41 | 1/41 | 7/40 | 5/41 | 2/47 |  |  |  |  |
| G371A |  |  |  |  |  |  | 3/47 | 10/35 | 5/39 |
| T399C |  |  |  |  |  |  | 1/47 |  |  |
| T494C |  |  |  |  |  |  | 1/47 |  |  |
| G500A |  |  |  |  | 1/47 |  |  |  |  |
| G524A |  |  |  |  |  |  |  | 1/35 |  |
| C525G |  |  |  |  |  |  | 1/47 |  |  |
| C556G |  |  |  |  |  |  | 1/47 |  |  |
| C562T |  |  |  |  |  |  | 2/47 |  |  |
| G646A | 2/41 |  |  | 2/41 |  |  |  |  |  |
| G711A |  |  |  |  |  |  |  | 1/35 |  |
| G711T |  |  |  |  |  |  |  | 1/35 |  |
| C720G |  |  |  |  |  |  |  | 3/35 | 8/39 |
| C720A |  |  |  |  |  |  |  |  | 3/39 |
| G721A |  | 33/41 | 27/40 |  | 35/47 | 29/45 |  |  |  |
| G726A |  |  |  |  |  |  | 1/47 |  |  |
| C95T  G298A |  |  |  |  |  |  | 1/47 |  |  |
| G149A  T207C |  |  |  |  |  |  | 1/47 |  |  |
| C90T  G371A |  |  |  |  |  |  |  |  | 1/39 |
| G149A  G371A |  |  |  |  |  |  | 1/47 |  |  |
| C157T  G371A |  |  |  |  |  |  | 1/47 |  |  |
| G191A  G371A |  |  |  |  |  |  |  |  | 1/39 |
| C525G  G371A |  |  |  |  |  |  |  |  | 1/39 |
| G371A  A594G |  |  |  |  |  |  | 1/47 |  |  |
| A675G  A701G |  |  |  |  |  |  | 1/47 |  |  |
| A61G  C720G |  |  |  |  |  |  |  |  | 1/39 |
| C74T  C720A |  |  |  |  |  |  |  |  | 3/39 |
| C74T  C720G |  |  |  |  |  |  |  |  | 1/39 |
| C90T  C720A |  |  |  |  |  |  |  |  | 1/39 |
| G2A  G721A |  |  |  |  |  | 1/45 |  |  |  |
| T9A  G721A |  |  |  |  |  |  |  |  |  |
| G37A  G721A |  |  |  |  | 1/47 |  |  |  |  |
| G54A  C344T |  |  |  |  |  |  |  |  |  |
| G371A  C525T |  |  |  |  |  |  |  | 1/35 |  |
| G54A  G721A |  |  |  |  |  |  |  |  |  |
| C55T  G721A |  |  |  |  | 1/47 |  |  |  |  |
| C64T  G721A |  | 1/41 |  |  |  |  |  |  |  |
| G81T  G721A |  |  |  |  |  | 7/45 |  |  |  |
| C117G  G721A |  |  |  |  |  |  |  |  |  |
| T172G  G721A |  | 1/41 |  |  |  |  |  |  |  |
| G195C  G721A |  |  |  |  |  | 1/45 |  |  |  |
| C206T  G721A |  |  | 1/40 |  |  |  |  |  |  |
| C230G  G721A |  | 1/41 |  |  |  | 1/45 |  |  |  |
| C255T  G721A |  |  | 1/40 |  |  |  |  |  |  |
| C296T  G721A |  |  |  |  |  |  |  |  |  |
| C327T  G721A |  |  |  |  |  | 1/45 |  |  |  |
| C344T  G721A |  | 1/41 |  |  |  | 2/45 |  |  |  |
| T393C  G721A |  |  |  |  | 1/47 |  |  |  |  |
| C457A  G721A |  |  |  |  |  | 1/45 |  |  |  |
| G593A  G721A |  |  |  |  |  |  |  |  |  |
| C623T  G721A |  |  |  |  |  |  |  |  |  |
| G652A  G721A |  |  |  |  |  | 1/45 |  |  |  |
| G721A  A758G |  |  |  |  | 1/47 |  |  |  |  |
| G363T  G371A  C405T |  |  |  |  |  |  | 1/47 |  |  |
| C90T  G371A  G543A |  |  |  |  |  |  |  | 1/35 |  |
| G291C  G371A  G383C |  |  |  |  |  |  |  |  | 1/39 |
| G33T  G36A  G721A |  |  |  |  |  |  |  |  |  |
| G33T  C55T  G721A |  | 1/41 |  |  |  |  |  |  |  |
| C55T  G193A  G195A  G721A |  |  |  |  |  |  |  |  |  |
| the type of mutations | 2/41 | 8/41 | 4/40 | 2/41 | 7/47 | 10/45 | 21/47 | 8/35 | 10/39 |
| the total number of mutations | 7 | 46 | 38 | 7 | 47 | 59 | 29 | 21 | 39 |
| the total number of bases (bp) | 31488 | 31488 | 30720 | 31488 | 36096 | 34560 | 36096 | 26880 | 29952 |
| Frequency per 10^4^ | 2.22 | 14.61 | 12.37 | 2.22 | 13.02 | 17.07 | 8.03 | 7.81 | 13.02 |

the type of mutations: 2/41 represents 2 mutant cells in 41 successfully sequenced clones;

the total number of bases: represents the total number of bases corresponding to the target gene in the successfully sequenced clones, eg: 41×768bp=31488bp;

Frequency per 10^4^: the total number of mutations/ the total number of bases;

R1、R2 、R3 respectively represent 3 rounds of evolution.

CDRs are highlighted in colors (HC-CDR1, HC-CDR2, HC-CDR3, LC-CDR1, LC-CDR2, LC-CDR3).

Figure S2


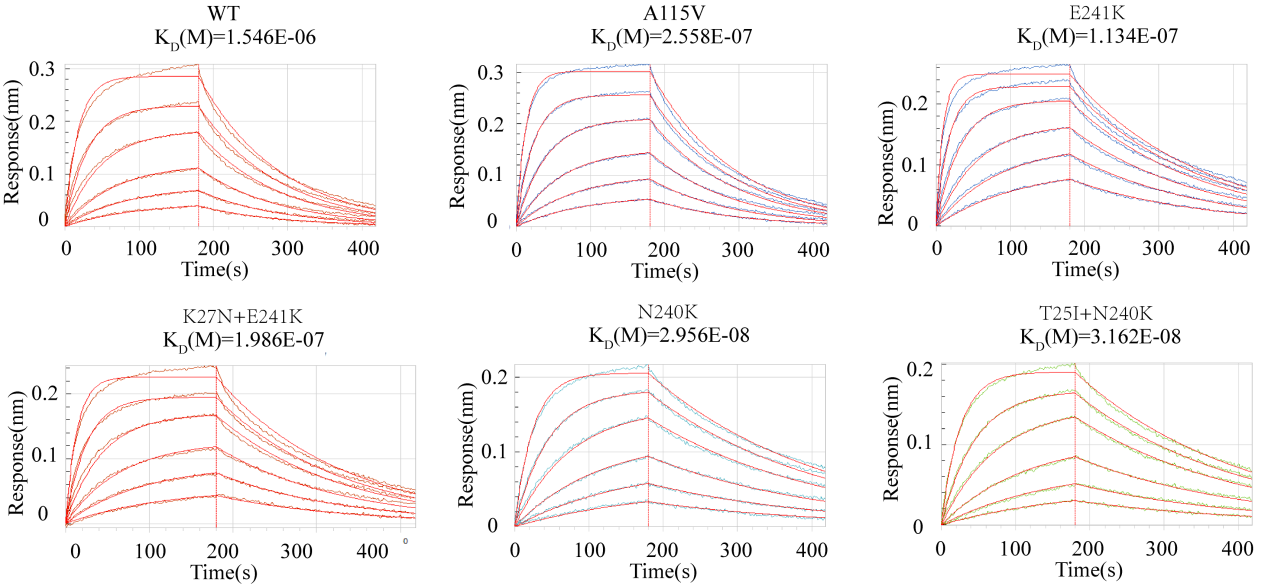


Figure S2 The results of antibody affinity. The basic procedure was to immobilize the biotinylated antigen on a chip, and the antibody acted as a mobile phase to detect the affinity of the antibody. K_on_ and K_off_ were obtained by the system software based on the combination and dissociation curves, while the K_D_ values were calculated by K_off_ / K_on_.
